# Perineuronal Nets and Metal Cation Concentrations in the Microenvironments of Fast-Spiking, Parvalbumin-Expressing GABAergic Interneurons: Relevance to Neurodevelopment and Neurodevelopmental Disorders

**DOI:** 10.3390/biom11081235

**Published:** 2021-08-18

**Authors:** Jessica A. Burket, Jason D. Webb, Stephen I. Deutsch

**Affiliations:** 1 Department of Molecular Biology & Chemistry, Christopher Newport University, Newport News, VA 23606, USA; jessica.burket@cnu.edu (J.A.B.); jason.webb.17@cnu.edu (J.D.W.); 2 Department of Psychiatry and Behavioral Sciences, Eastern Virginia Medical School, 825 Fairfax Avenue, Suite 710, Norfolk, VA 23507, USA

**Keywords:** perineuronal net, iron, copper, fast-spiking, parvalbumin, autism spectrum disorder

## Abstract

Because of their abilities to catalyze generation of toxic free radical species, free concentrations of the redox reactive metals iron and copper are highly regulated. Importantly, desired neurobiological effects of these redox reactive metal cations occur within very narrow ranges of their local concentrations. For example, synaptic release of free copper acts locally to modulate NMDA receptor-mediated neurotransmission. Moreover, within the developing brain, iron is critical to hippocampal maturation and the differentiation of parvalbumin-expressing neurons, whose soma and dendrites are surrounded by perineuronal nets (PNNs). The PNNs are a specialized component of brain extracellular matrix, whose polyanionic character supports the fast-spiking electrophysiological properties of these parvalbumin-expressing GABAergic interneurons. In addition to binding cations and creation of the Donnan equilibrium that support the fast-spiking properties of this subset of interneurons, the complex architecture of PNNs also binds metal cations, which may serve a protective function against oxidative damage, especially of these fast-spiking neurons. Data suggest that pathological disturbance of the population of fast-spiking, parvalbumin-expressing GABAergic inhibitory interneurons occur in at least some clinical presentations, which leads to disruption of the synchronous oscillatory output of assemblies of pyramidal neurons. Increased expression of the GluN2A NMDA receptor subunit on parvalbumin-expressing interneurons is linked to functional maturation of both these neurons and the perineuronal nets that surround them. Disruption of GluN2A expression shows increased susceptibility to oxidative stress, reflected in redox dysregulation and delayed maturation of PNNs. This may be especially relevant to neurodevelopmental disorders, including autism spectrum disorder. Conceivably, binding of metal redox reactive cations by the perineuronal net helps to maintain safe local concentrations, and also serves as a reservoir buffering against second-to-second fluctuations in their concentrations outside of a narrow physiological range.

## 1. Introduction

There are many proteins participating in binding, transport, and distribution of the redox reactive metals iron and copper throughout the body and across membranes, including across neuronal cell membranes and those of specialized organelles within cells. These metal cations are also crucial to the catalytic function of many enzymes and mitochondrial respiration. For example, synaptic release of free copper also acts locally to modulate neurotransmission [1]. Within the developing brain, iron is critical for hippocampal development and the differentiation of parvalbumin-expressing neurons whose soma and dendrites are surrounded by perineuronal nets (PNNs), a specialized elaboration of the extracellular matrix [2,3,4]. Free concentrations of the redox reactive metals iron and copper are highly regulated because of their abilities to catalyze generation of toxic free radical species. The complex architecture of PNNs bind metal cations, which serves a protective function against oxidative damage, especially oxidative damage of the fast-spiking neurons with their dense concentration of rapidly respiring mitochondria [5,6,7,8]. It is conceivable that the binding of these metal redox reactive cations not only contributes to maintenance of their safe local concentrations, but also serves as a reservoir that can “buffer” against second-to-second fluctuations in their concentrations and availability [4].

**Figure 1:**
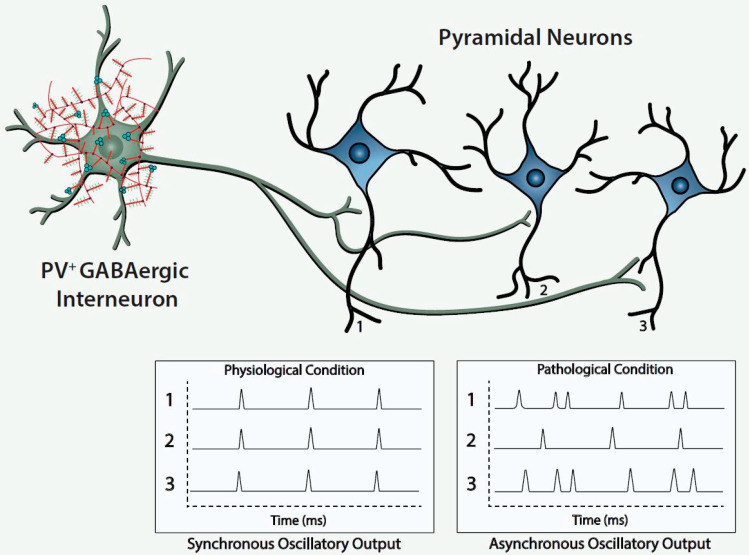
Regulation of synchronous oscillatory activity of pyramidal cell neurons by GABAergic interneurons.

Histofluorescent-staining of PNNs with specific plant lectins is a useful morphologic approach to identify subpopulations of fast-spiking, parvalbumin-expressing (FSPV+) GABAergic interneurons, as well as populations of pyramidal neurons. Within the cerebral cortex and hippocampus, fast-spiking, PV-expressing GABAergic interneurons are the most populous neurons enwrapped by PNNs, which is consistent with an important role of the PNN in maintaining and protecting energy-efficient and rapid information transfer. These interneurons regulate the synchronous oscillatory output of assemblies of pyramidal output neurons that are computed “upward” into gamma-band synchrony of 30–80 Hz [4,9,10,11]. Gamma-band synchrony across cortical areas facilitates functional connectivity between them and is necessary for performance of many higher cognitive functions, such as working memory, response selection, object representation, attention and sensorimotor integration, among other functions [4,9,10,12,13,14]. Disturbance of these fast-spiking, parvalbumin-expressing neurons leads to disruption of the synchronous oscillatory output of assemblies of pyramidal neurons that ultimately impact functional connectivity and higher cognitive functions (Figure 1). 

The figure depicts a fast-spiking parvalbumin-expressing GABAergic projection onto an assembly of neocortical pyramidal output neurons. The inhibitory input of the GABAergic projection synchronizes the oscillatory output of the assembled pyramidal neurons, which, in turn, is computed upward into rhythms that may mediate functional connectivity across disparate regions of the cerebral cortex. Conceivably, abnormalities of the handling of redox-reactive metals by the PNNs surrounding the neurons in this circuit would lead to impairment of the fast-spiking capability of the GABAergic projection and/or oxidative damage to the GABAergic projection or pyramidal neurons comprising this neocortical circuit. This pathological disturbance is depicted in the figure by the asynchronous firing of the pyramidal neurons. Ultimately, disruption of fast-spiking and/or oxidative damage would lead to dysrhythmias that impair functional connectivity.

From a neurodevelopmental perspective, morphologically mature PNNs are implicated in the closing of critical periods, which may involve actual obstruction to the entry of neuritic extensions. Thus, therapeutic disruption of mature PNNs is proposed as a mechanism for promoting plasticity in some neurodevelopmental disorder [4]. Neurobiological interest in numerous functional roles of the PNNs themselves continues to grow and the possible contributions of neocortical circuits, comprised of projections of fast-spiking, parvalbumin-expressing GABAergic inhibitory neurons to assemblies of pyramidal neurons, to the pathogenesis of schizophrenia and autism spectrum disorder [3,4,15,16]. Functionally, the PNNs are implicated in the stabilization of synapses and consolidation of long-term memory [4,17,18]. The maturation of the PNN is also implicated in the mechanism of closing critical periods of neural circuit development, creating a physical barrier, thereby reducing neural plasticity [6,14,19]. Although somewhat paradoxical and, perhaps, counter-intuitive, impeding plasticity may assure energy-efficient and more rapid information transfer within dedicated circuits, reflecting selection of shortest path lengths within circuits that are “quiet” and shielded from interference by extraneous “noise”. The current review seeks to provide a brief overview of PNN structure and function and explore a new and emerging area of interest related to the regulation of local concentrations of critical redox metal ions and possible functional consequences and therapeutic potential of their interaction and regulation with PNNs.

## 2. Architecture and Function of Perineuronal Nets and their Structural Components

**Figure 2:**
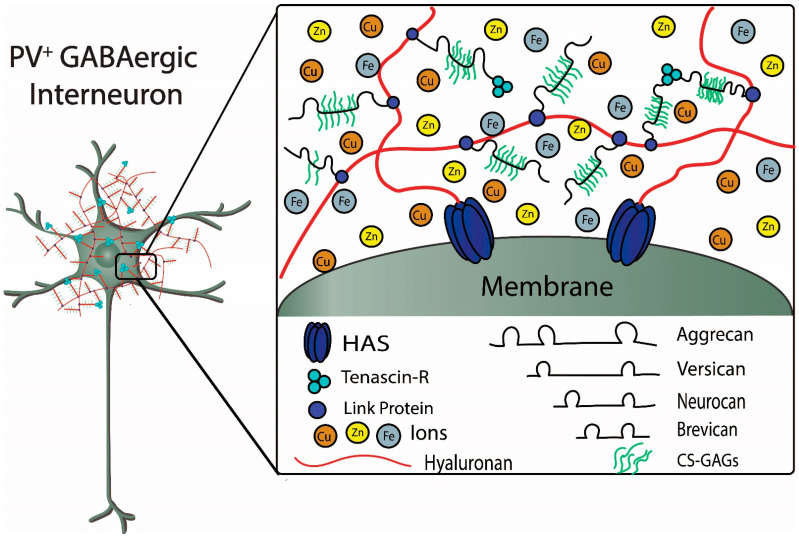
Components of the perineuronal net (PNN) and interaction with metal ions.

PNNs are large, specialized, stable meshwork structures that surround cell bodies and proximal dendrites of subpopulations of neurons. PNNs are composed of chondroitin sulfate proteoglycans (CSPGs) that include lecticans, such as aggrecan and brevican, connected to a long hyaluronan backbone, which is stabilized and cross-linked by linkage proteins, such as cartilage link protein (Crtl-1/HAPLN-1) and brain link protein (Bral2/HAPLN-4), and tenascin-R (TN-R) [4,20,21,22,23,24,25] (Figure 2). CSPGs are secreted by a variety of cell types and incorporated into PNNs. Within the Golgi apparatus, the repeating chondroitin sulfate disaccharide unit is attached to the core protein via three sequential sugars, and the activity of chondroitin synthase determines the length of the repeating chondroitin sulfate glycosaminoglycan (GAG). Importantly, the properties of the CSPGs are determined by the specific core protein, extent of glycation (length and number of GAG side chains), and patterns of sulfation. The type and distribution of CSPGs within the CNS are not uniform; for example, sulfation of N-acetylgalactosamine in both the C4 and C6 positions, referred to as CS-E, is commonly found in cerebral cortex, whereas in cerebellum, a common sulfation pattern is the C6 position of N-acetylgalactosamine and the C2 position of N-glucuronic acid, referred to as CS-D [14,23,26,27]. Based on their physico-chemical properties, the dense polyanionic microenvironment created by the long hyaluronic acid backbone and sulfated GAG side chains of the PNN are thought to maintain the fast-spiking properties of GABAergic inhibitory interneurons expressing parvalbumin (PV), a low-affinity, high-capacity Ca^2+^-binding protein, and protect against iron-induced oxidative stress and damage [22,28]. 

The figure depicts structural components of a PNN surrounding a fast-spiking parvalbumin-expressing GABAergic inhibitory neuron. Metal ions interacting with components of the PNN are also depicted. Chondroitin sulfate proteoglycans (CSPGs), which include aggrecan, versican, neurocan, and brevican, are connected to the hyaluronan backbone that is synthesized by the hyaluronan synthases (HAS). The CSPGs are connected and stabilized to hyaluronan by link proteins. Chondroitin sulfate glycosaminoglycans (CS-GAGs) are repeating disaccharide units that bind to the core proteoglycan proteins. The sulfation patterns of the GAGs determine binding and diffusion properties of cations and metal ions within the PNNs. Tenascin-R forms a trimeric complex that contributes to stabilization of the PNN by connecting multiple CSPGs (see text for further details) (Figure adapted from Tsien, RY 2013).

Lectican is the general term to refer to the most commonly expressed CSPGs in CNS; aggrecan, versican, neurocan, and brevican are examples of lecticans found in PNNs. Additionally, lecticans are found in the nodes of Ranvier where they interact with astrocytes (e.g., versican and brevican) and are also found at synapses (e.g., brevican) where they may interact with both pre- and post-synaptic molecules, including receptors, such as the AMPA (α-Amino-3-hydroxy-5-methyl-4-isoxazolepropionic acid) receptor [14,15,24,25]. Aggrecan is found most frequently in PNNs, often serving as an immunohistochemical marker to identify their presence. Aggrecan has the greatest density of GAG side chains and its core protein has an additional N-terminal domain, when compared with the other lecticans. The globular N- and C-terminals serve as linkage sites for anchoring the GAG side chains to hyaluronan and tenascin-R, respectively, and their domain structures may also have functional significance. The N-terminal globular domain, referred to as G1, has two distinct structures that are cross-linked with each other via the disulfide bonding of conserved cysteine amino acids within each of them: an IgG-like loop and a link protein-like proteoglycan tandem repeat (PTR) similar to those coded by the “HAPLN” family of genes for link proteins [14,29,30]. As noted, aggrecan also contains an additional N-terminal G2 domain that contains the PTR structure. The C-terminal G3 domain of the lecticans contains EGF and complement regulatory protein (CRP)-like domains [14].

In terms of the structural significance of these domains, the 50 kDa cartilage link protein 1 (HAPLN1) interacts with the G1 domain of aggrecan to create a stable, non-covalently linked aggrecan to the hyaluronan backbone. Thus, when the link proteins HAPLN1 and HAPLN4 bind the lecticans to the hyaluronan backbone, a tripartite complex is formed between the N-terminal G1 domain of lecticans, hyaluronan and a HAPLN link protein. HAPLN1 and HAPLN4 are found in PNNs, whereas HAPLN2 is made by oligodendrocytes and associated with the nodes of Ranvier interacting with versican. Hyaluronan is a non-sulfated GAG that is made on the surface of the cell by three different isoforms of membrane-bound hyaluronan synthase (HAS), each of which produces different lengths at different rates [14,31]. As noted, the C-terminal of the lecticans bind to tenascin-R. Interestingly, a recent in vitro study examined electrophysiological alterations in the excitatory and inhibitory ratio of hippocampal neurons in a quadruple knockout of PNN components (e.g., tenascin-C, Tenascin-R, neurocan, and brevican), supporting the role of PNNs in synaptogenesis [32].

### 2.1. Structural Components of PNNs Scavenge and Bind Metal Ions: Protective Effects against Oxidative Damage

**Figure 3:**
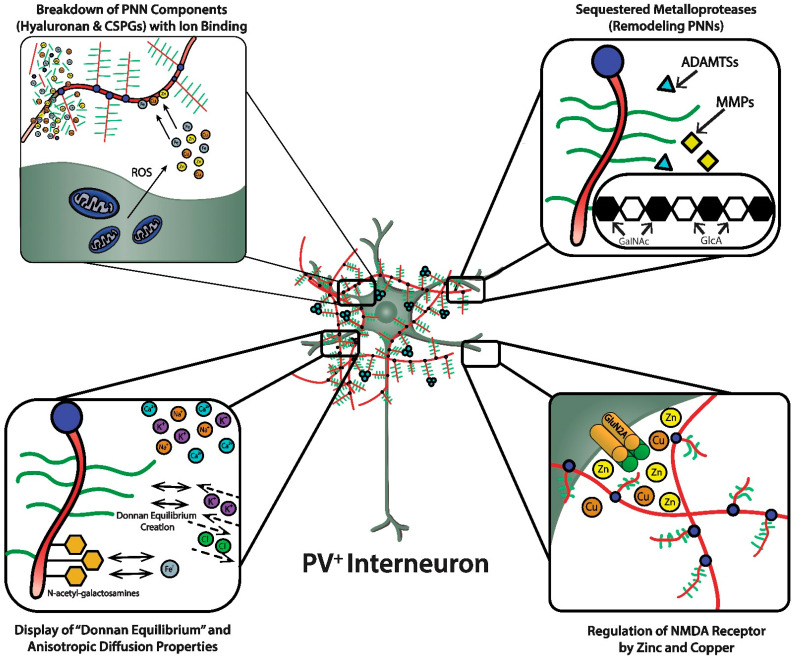
Functional roles of perineuronal nets (PNNs).

Hyaluronic acid (HA), a long linear polysaccharide composed of repeating alternating disaccharide units of D-glucuronic acid and N-acetyl-D-glucosamine that adopts a three-dimensional structure in solution, is a major constituent of extracellular matrix (ECM) and the backbone of the specialized PNN [2,4,33]. The long polysaccharide chain of HA undergoes extensive intramolecular hydrogen bonding that conveys gel-like properties to aqueous solutions due to increased viscosity that affects diffusion properties, especially those of charged solutes [4]. The polyanionic character of HA is due to the negatively charged free carboxyl groups of the D-glucuronic acid throughout the length of the polysaccharide. The binding of bivalent Fe^2+^ to two carboxyl groups of a tetrasaccharide unit leads to the generation of reactive oxygen species (ROS), including the hydroxyl radical and superoxide anion, and peroxynitrite, a reactive nitrogen species (RNS), and oxidative degradation of HA [33]. Importantly, Cu^2+^ can actually have a protective effect against the oxidative degradation of HA initiated by Fe^2+^; this protective effect is, in large part, due to the ability of Cu^2+^ to displace the bound Fe^2+^ from HA. There is also interest in the ability of bivalent metal ions to provide protection by scavenging reactive free radicals independently of binding to HA [33]. On a purely physicochemical basis, the polyanionic character of the long HA polysaccharide chain attracts and binds metal ions, which, in the cases of Cu^2+^, Co^2+^, and Zn^2+^, may play important roles in protection against oxidative damage to HA, in addition to scavenging highly reactive free radicals (Figure 3, upper left panel). The integrity of the HA polysaccharide chain in the ECM and PNN is important because of the viscosity it conveys to the aqueous medium that affects critical biological processes during brain development and beyond, such as cell adhesion, migration, and proliferation [14,33]. Of course, potential beneficial antioxidant effects are only observed within narrow concentration ranges; thus, the evolutionary selection of proteins that bind, transport and use these metal cations as cofactors for precisely controlled catalytic functions, and maintain their free concentrations within narrow and safe ranges. 

PNNs were originally viewed as a curious morphological artifact, but are now recognized to be highly specialized elaborations of the extracellular matrix (ECM). PNNs are shown as densely-negatively charged lattice structures that enwrap subpopulations of GABAergic inhibitory neurons. The upper-left magnified box depicts the polyanionic hyaluronan and CSPG lattice-like structure and their ability to bind and create a reservoir of metal cations. This magnified box also shows (upper-left) the dense concentration of mitochondria in these fast-spiking, parvalbumin-expressing inhibitory interneurons that are necessary to maintain their fast-spiking properties, but also generate reactive oxygen species. PNNs are critical for maintaining fast-spiking properties of the subset of GABAergic inhibitory interneurons they enwrap via providing a reservoir of cations and creating a “Donnan Equilibrium” (lower-left; see text for details) that influence the directional movement of critical ions and metals, in addition to serving as an “ionic filter”, and structure that sequesters metalloproteinases and transcriptional factors (upper-right; see text for details). The current review discusses their involvement in maintaining a safe “redox environment”. For example, anionic binding sites within the PNN help to maintain nontoxic concentrations of redox-relevant metal ions in the local environments of the neurons that they enwrap. The lower-right magnified box depicts the binding of Zn^2+^ and Cu^2+^, metal ions that modulates the excitability of NMDA receptors. Perineuronal nets participate in regulating concentrations of metal ions that influence the activity and metabolism of the neurons they enwrap.

The profile of “Selective Neuronal Vulnerability” of different neurons and brain areas to neurofibrillary degeneration is used in neuropathological staging of Alzheimer’s disease (AD) [22]. PNNs bind and scavenge ‘redox active iron”, which is their proposed mechanism of protection against oxidative damage. Consistent with this proposed mechanism of protection, accumulation of lipofuscin within neurons of patients with AD, an intralysosomal pigment generated by iron-catalyzed oxidation, is reduced in those neurons surrounded by PNNs, compared to neurons not surrounded by PNNs. Interestingly, cortical and subcortical neurons enwrapped by PNNs in AD brain are protected against neurofibrillary degeneration. This was demonstrated in a histochemical study that sought to confirm that PNNs are protective against iron-induced oxidative damage using 3-month-old knockout mice with deletions of genes encoding critical PNN structural components to identify if these selectively knocked-out components were necessary for, or contributed to, antioxidant effects [22]. 

Specifically, neurodegenerative effects of iron were studied in knockout mice homozygous for deletion of brevican (BCAN-/-), tenascin-R (TN-R-/-), and cartilage link protein 1 (HAPLN1-/-) and heterozygous for deletion of aggrecan (ACAN+/-) [22]. Neurodegeneration was accessed 24 h after mice received stereotactic injections of either FeCl3 or NaCl control into the barrel field of the somatosensory cortex by identifying and distinguishing neurons ensheathed in PNNs to those neurons without PNNs [22]. Importantly, in the wild-type mice, about 10% of the neurons detected in the barrel field cortex were enwrapped in PNNs and 49% of these neurons enwrapped by PNNs expressed PV [22]. The morphological appearance of PNNs did not differ noticeably in the knockout mice with homozygous and heterozygous deletions of brevican and aggrecan, respectively, however, PNN morphology of mice lacking complete expression of cartilage link protein 1 (HAPLN1-/-) and tenascin-R (TN-R-/-) was markedly altered from the wild-type mice [23]. The PNN morphology of HAPLN1-/- and TN-R-/- mice was described as more fuzzy and granular with reduced staining around dendrites [22]. Irrespective of reductions in expression of PNN components, the total numerical density of neurons did not differ between wild-type mice and any of the knockout strains. Further, absent expression of brevican and haploinsufficient expression of aggrecan had no effect on the number of neurons enwrapped by PNNs. However, a reduction in the number of neurons enwrapped by PNNs and the number of PV-expressing neurons enwrapped by PNNs, in particular, were seen in HAPLN1-/- and TN-R-/- mice. Moreover, absent expression of TN-R-/- and the altered morphology of PNNs in these knockout mice were associated with a lowered percentage of PV-expressing neurons enwrapped with PNNs compared to neurons enwrapped with PNNs but not expressing PV. Relative to wild-type, the size of the iron (FeCl3)-induced oxidatively-damaged neurodegenerative lesion was significantly greater in all of the knockout strains with reduced or absent expression of components of the PNNs, including haploinsufficient expression of aggrecan, and absent expression of brevican, cartilage link protein 1, and tenascin-R [22]. 

When double-labeling histochemical staining procedures were used to examine markers of degeneration and PNNs simultaneously in single cells, the protective effects of PNNs against neurodegeneration appeared to be abolished in the HAPLN1-/- and TN-R-/- knockout mice. Moreover, when triple-labeling procedures were employed to explore effects of PV-expression on iron-induced oxidative damage in PNN-ensheathed neurons in wild-type and knockout mouse strains, expression of PV by the neuron did not increase protection against neurodegenerative effects of iron. In fact, when comparing BCAN/- mice, there was more frequent neurodegeneration observed in those PNN-ensheathed neurons immunoreactively-positive for expression of PV than found among the PNN-enwrapped neurons showing no immunostaining for PV. Interestingly, aggrecan expression reflected an interaction between genotype and iron (FeCl3)-induced oxidative stress. Specifically, increased immunoreactive aggrecan was observed on the side of the stereotactic infusion of FeCl_3_ into the barrel field in HAPLN1-/- and BCAN-/- mice compared to the contralateral control side [22]. A posited role of PNNs includes sequestration of a transcriptional factor(s) within its meshwork lattice that may be transported into and, thereby, influence selective gene expression within the PV-expressing GABAergic interneurons they surround [4,34]. Clear disruption of the PNN’s neuroprotective properties against iron-induced oxidative damage were associated with absent expression of the cartilage link protein 1 and tenascin-R [22]. Thus, the morphologically complex lattice meshwork that surrounds cell bodies and proximal dendrites is a necessary requirement for maximal protection against iron-induced oxidative damage. Aggrecan, in conjunction with the hyaluronan backbone, contributes significantly to the polyanionic character of the PNN. Interestingly, aggrecan expression was selectively upregulated 24 h after injection of iron in the knockout mice lacking expression of either cartilage link protein 1 or brevican.

### 2.2. Regulation of Sulfation Patterns within PNNs Contribute to Calcium Chelation and Transmembrane Potential and Excitability

As described above, chondroitin sulfates (CSs) are repeating disaccharide units of D-glucuronic acid β (1-3) and D-N-acetylgalactosamine β (1-4) making up the glycosaminoglycan side chains (GAGs) that bind to core proteoglycan proteins (PGs) [35]; examples of these structures, referred to as CSPGs, include aggrecan and brevican. The lengths of the GAGs and their sulfation patterns within the brain’s ECM and its specialized PNNs influence their interactions with diverse molecules, such as growth factors and chemokines, and the transmembrane potentials of excitable neuronal membranes via their effects on the extracellular free calcium ion concentration in the local microenvironments of neurons [35]. These interactions and influences of PNNs are explained by the physicochemical properties of the CSPGs and the hyaluronan backbone and their complex patterns of linkages and cross-linkages [35,36]. For example, chain length and branching, electronic architecture, and density of negatively charged sulfate and carboxylic acid residues and the pattern of sulfation determine binding and diffusion properties of the calcium ion and ionic metals, such as iron and copper, within the brain’s ECM. Ionic metals do not diffuse freely and there is an anisotropic pattern to their diffusion [36].

Importantly, there is a dynamic remodeling of GAGs and CSPGs mediated by proteases, glycosidases and sulfatases, whose activities are modulated in response to physiological and pathological processes [14,35]. Sulfation can occur at the C4 and C6 positions of N-acetylgalactosamine and the C2 position of D-glucuronic acid; again, the patterns of sulfation are neither invariant nor random and can have consequences for the free extracellular calcium ion concentration and transmembrane potential. Purified solutions of chondroitin 4 sulfate (CS4) and chondroitin 6 sulfate (CS6) were used to measure their calcium ion binding properties [35]. Very briefly, the construction of chelation curves to measure the binding capabilities of free calcium ions involved incubating purified solutions of CS4 and CS6 with solutions of free calcium. Hyaluronan lacks sulfation and, thus, was shown to have limited calcium ion binding capability. Bound calcium ions were dissociated from the purified chondroitin sulfate preparations, and the amount of dissociated bound calcium ion was measured. The functional significance of differences in the binding capabilities of CS4 and CS6 with calcium ions was studied with electrophysiological recordings from light-adapted principal rods of single cells; photoreceptor suspensions were prepared from retina of *Xenopus laevis*. 

The data showed that CS4 bound significantly more calcium ion than CS6 when incubated with a fixed concentration of calcium ions (1 mM) [35]; thus, the sulfation position exerts an effect on the calcium chelating capabilities of the CS. Moreover, the sulfation patterns of the CSPGs affect charge separation and current flow of free Ca^2+^ ions across the membrane. Calcium ion current flow through voltage-gated ion channels was studied over a range of clamped membrane potentials (i.e., the relationship between conductance changes as a function of voltage was plotted). In this way, the abilities of CS4 and CS6 added to the perfusate containing a fixed 1 mM concentration of CaCl_2_ to shift the control curve to the left by virtue of its calcium ion chelating properties were assessed. Using the patch-clamp technique, the calcium ion chelating property of CS4 was shown to affect current flow across ‘voltage-operated ion channels (VOC)’ significantly by reducing concentrations of free calcium ions in the perfusate [35]. CS4 was able to significantly shift the control curve to the left, but its slope factor (k), which indicates the steepness of the voltage dependence, did not differ significantly from the control curve; this was reflected in a similar shape describing the relationship between conductance and voltage between the CS4 and control curves. The addition of CS6 was also able to shift the control curve to the left but the slope factor differed significantly from the control curve and the shape of its conductance-voltage relationship was dissimilar to that of both the CS4 and control curves [35]. These data suggest that CSs exert influence on transmembrane potential. Through a local effect on extracellular concentrations of free calcium ions at the surface of the outer cell membrane, whose membrane phospholipids carry a net negative charge that can serve to “screen” these divalent cations, the voltage sensors of the voltage-gated ion channels may detect changes in average voltage difference or voltage drop across the membrane and, thereby, trigger changes in conductance [35]. In any event, sulfation patterns of the CSs and local differences in the ratios of CS4 to CS6 in the ECM and PNN have functional implications for excitability and, ultimately, information transfer within neural circuits.

### 2.3. Ion Sorting, Anionic Shielding, and Donnan Equilibrium Properties Protect against Oxidative Damage and Maintain the Rapid-Firing of Fast-Spiking, PV-Expressing Neurons

Reduced states of redox cycling iron and copper are oxidized transferring single electrons to molecular oxygen (O_2_) and hydrogen peroxide (H_2_O_2_) to produce the superoxide anion and the hydroxyl free radical, respectively. ROS have the potential to damage carbohydrates, lipids, proteins and DNA. Excess concentrations of zinc can cause leakage of electrons from mitochondria and, in addition, through zinc’s activation of protein kinase C and NADPH oxidase and induction of nitric oxide synthase can also lead to formation of ROS and RNS. Although antioxidant defenses, including Cu/Zn superoxide dismutase (SOD) in the cytosol and mitochondrial intermembrane space and Mn SOD in the mitochondrial matrix, and metal chaperones and transport and storage proteins exist to keep metals in a nontoxic and bioavailable form, these defenses can be overcome, especially in areas of brain with exceptionally dense concentrations of mitochondria engaged in active respiratory chain activity [4,37,38]. In addition to PNN’s ion sorting, anionic shielding and Donnan equilibrium properties, SOD binds to GAG side chains on the PNN CSPGs and can serve as an additional barrier of protection against oxidative damage caused by increased concentrations of metal cations in the local microenvironment of actively respiring neurons [4,28,39,40].

As discussed, the GAG components of the PNNs are responsible for dense negative charges in the microenvironment of the subset of neurons they ensheath [36]. These fixed anionic binding sites influence diffusion properties of charged diffusing particles and acts as an immobilized ion exchanger, which partitions ions according to their charge and Donnan equilibrium (Figure 3, lower left panel). Using a novel approach (i.e., “high resolution nuclear microscopy with focused proton-beam microprobes in combination with an ionic metallic probe”), fixed negative charge density in the neuronal microenvironment were visualized and quantified in brain slices; also, the methodology enabled measurement of bound cationic probes, such as Fe^3+^ ions, within PNNs [36]. Relative amounts of iron atoms per sulfur atoms in PNNs varied according to brain region, ranging from a high of ~3.8 in the subiculum to a low of ~1.3 in the red nucleus. This binding reflects the relative amount of iron ions bound to the chondroitin sulfate disaccharide units because iron labeling disappeared when brain sections were treated with chondroitinase or hyaluronidase, which removes GAG side chains. Moreover, the specialized PNNs accumulated two to three times more Fe^3+^ than ECM in general. The anionic charge density of the PNNs afforded estimates of the maximal binding of Fe^3+^, which were in the 117 to 180 mM range (which are much higher estimates than previously suspected). Diffusion properties in regions enriched with PNNs are anisotropic, reflecting their interference with free diffusion. 

In addition to hindering free diffusion of charged particles, PNNs create an extracellular reservoir of Ca^2+^, K^+^, and Na^+^, and the Donnan equilibrium provides an inward directed force on Cl^−^ and an outward directed force on K^+^ from the perspective of the ensheathed neuron, which supports fast-spiking properties (Figure 3, lower left panel). Thus, the presence of PNNs with their dense negative charge contributes to the anisotropic movement of ions and charged particles, preventing random distribution of these ions and charged particles in the vicinity of the PNN.

Finally, the PNN creates an “anionic shield” that protects neurons they ensheath from toxic anionic species, such as hydroxyl ions [OH]^−^. The anionic shield protects against a variety of “highly reactive” hydrated electron species [36]. Thus, PNNs, a morphological elaboration of the ECM that was identified in early neurohistological studies (and dismissed by some as an unimportant curiosity), are postulated to have many critical functional roles, especially in support of the subpopulation of neurons they ensheath [4,5,41]. 

### 2.4. Dynamic Remodeling of PNNs: Potential Therapeutic Role for Selective Inhibitors of Remodeling Enzymes

A complex enzymology is involved in the dynamic remodeling of PNNs that almost certainly affects learning, memory and efficiency of information transfer within neural circuits; moreover, their remodeling may be part of pathogenic mechanisms in a variety of CNS disorders [3,14,15,42]. In fact, there is already therapeutic interest in selective inhibitors of several of these remodeling enzymes. In addition to functions attributed to their physicochemical properties, such as ion sorting, anionic shielding, and the impact of the Donnan equilibrium on rapid adjustments of membrane potential, the three-dimensional scaffolding structure of PNNs in particular, and the ECM in general, affects signal transduction through their interactions with molecules on the cell surface, including integrins and cadherins, brain development through regulation of cell migration, and both normal and pathological aging [14]. 

A specific ‘matrix metalloproteinase (MMP)’, referred to as MMP-9, a member of a family of zinc-containing endopeptidases, is involved in ECM remodeling. MMP-9 protein levels and activity are increased with induction of “long-lasting long-term potentiation (LTP)” [4,14,43]. Hippocampal LTP is altered by a variety of strategies that antagonize MMP-9, such as broad spectrum MMP inhibitors, antisense oligonucleotides, and neutralizing antibodies. A confluence of studies show complex relationships between LTP-inducing stimuli, MMP-9 release, and enlargement of dendritic spines. Moreover, the activity of MMP-9 and its relationship to LTP induction appears to undergo homeostatic regulation in the prefrontal cortex of rats via overexpression of a specific “tissue inhibitor of MMPs (TIMP)”, referred to as TIMP-1. The relations between plasticity, dendritic spine shape and density, expression and activity of MMP-9, and expression of TIMP-1 are complex and depend on the experimental preparation (e.g., tissue slice or cell culture), brain region (e.g., barrel cortex, visual cortex, hippocampus, or amygdala) and the nature and goal of the stimulation (e.g., LTP-induction, seizure induction, environmental enrichment, or sensory deprivation). There is transcriptional regulation of MMP-9 and TIMP-1 by activator protein 1 (AP-1), a transcriptional factor implicated in plasticity, and translational regulation of MMP-9 expression in the dendrite by the fragile X mental retardation protein (FMRP). The ability of MMP-9 inhibitors to attenuate abnormalities of spine morphology and density observed in mice modeling fragile-X-syndrome (FXS) has aroused therapeutic interest in this strategy. Interestingly, although none of the components of the PNN are known substrates of MMP-9, genetic reduction of MMP-9 in developing *Fmr-1* knockout (KO) mice promotes PNN formation around PV-expressing neurons [14,44]. This observation and others have focused research interest in downstream mediators or consequences of MMP-9 activation. There is also emerging interest in MMP-3, which is thought to act upstream of MMP-9 and may play a role in the latter’s activation [14,45]. 

Cortical fast-spiking, PV-expressing neurons enwrapped in PNNs express three metalloproteases (i.e., ADAMTS-8, ADAMTS-15, and Neprilysin); moreover, a role for their expression in PNN remodeling is strongly supported by the fact aggrecan and versican are substrates of ADAMTS-8 and ADAMTS-15 [4,14,46] (Figure 3, upper right panel). Emerging evidence also suggests that “tissue plasminogen activator (tPA)”, a member of the serine proteinase family that is better known for its therapeutic role in thrombotic stroke, may be involved in plasticity and ECM remodeling [14,47,48]. Hippocampal expression of tPA is induced by seizures and LTP and absence of its expression has adverse effects on LTP, spatial navigation tasks, and various learning paradigms. Lipoprotein receptor-related protein (LRP), the tPA receptor, is expressed in hippocampus and may mediate tPA’s effects on hippocampal LTP [14,49]. Downstream mechanisms may be responsible for effects of tPA on plasticity, such as its cleavage of pro-BDNF to BDNF and plasmin activation. Plasmin cleaves fibronectin and laminin components of the ECM. Although MMPs have pleiotropic effects that appear to have specificity with respect to brain region and disease process, “proof of principle/concept” preclinical and clinical studies of inhibitors suggest there may be some opportunities for therapeutic benefit. Novel compounds and repurposed medications with MMP-inhibitory activity, such as minocycline, doxycycline, and metformin, have been studied in diverse conditions, including experimental autoimmune encephalomyelitis (EAE), multiple sclerosis, ischemic stroke, and fragile X syndrome (FXS). Studies exploring selective small molecule inhibitors and genetic strategies to influence activity or expression of remodeling enzymes, respectively, will continue and resolve questions about the viability of targeting remodeling enzymes to treat CNS disorders in general, and autism spectrum disorder (ASD) in particular [4,50]. 

## 3. Iron Homeostasis, the Developing Brain, and Functional Consequences of Iron Handling by the PNN

Maternal iron deficiency during pregnancy and lactation in the human, especially during late gestation and through the third postnatal year, affects learning and memory in offspring [2,51]. Deficits, such as impaired facial recognition memory in childhood and impaired recognition memory at age 19, can persist into adulthood if iron replacement is delayed beyond the critical period of late gestation through the third postnatal year, which suggests a dependence of the hippocampus on adequate stores of available iron as it undergoes rapid development from late gestation through the first year of postnatal life. Restricting maternal intake of dietary iron in rodents causes iron deficiency in the offspring that is associated with impaired spatial learning (e.g., in the Morris water maze task) that is resistant to iron repletion if onset of restoration is delayed beyond a critical developmental date. Postnatal days (PD) 10 to 25 in rodents are a period of rapid hippocampal growth that is characterized by dense dendritic arborization, spine growth and synaptogenesis, as well as adult electrophysiological changes (e.g., in the CA1 region) [2,51]. 

Effects of iron deficiency on hippocampal development may reflect a more general epiphenomenon of iron deficiency (perhaps relating to relative hypoxia due to iron deficiency anemia at a time of increased demand and dependence of hippocampal pyramidal neurons on mitochondrial respiration), rather than a direct dependence of hippocampal development on adequate available local stores of iron [51]. Specifically, in order to better understand the effect of iron deficiency on hippocampal development, a reversible, dominant negative transferrin receptor-1 (DNTfR1) transgenic mouse model was created [51]. The normally-functioning transferrin receptor-1 (TfR1) binds diferric transferrin and is the first step in neuronal iron uptake. TfR1 is preferentially expressed on dendrites of hippocampal neurons. The DNTfR1 mouse model displays deficient iron uptake and storage in CA1 hippocampal pyramidal neurons and enabled reversible over-expression of nonfunctional DNTfR1 in these neurons. This model allowed for precisely timed manipulation of effective TfR1-mediated iron uptake in order to identify the critical period during which rapid hippocampal development is dependent on local uptake of available iron and permitted identification of times during which iron-replenishment could and could not reverse effects of iron-deficiency on spatial learning, CA1 dendritic arborization, and genetic expression of brain-derived neurotrophic factor (BDNF), an important plasticity factor [51]. Importantly, adequate availability of iron during a critical period of rapid hippocampal development was shown to be necessary for normal neurodevelopmental PV expression by a distinct subpopulation of neurons and their ensheathment by PNNs in the CA1 hippocampal region [2,51]. Expression of DNTfR1 in CHO cells cultured in vitro was associated with loss of transferrin binding capacity and iron uptake, compared to the normal transcript in this same cell line [51]. Moreover, in vivo studies confirmed that the DNTfR1 transcript was associated with reduced iron storage in the CA1 hippocampal region of these mice. Importantly, there was no reduction in hematocrit levels in the DNTfR1 mice suggesting that the pathologic changes observed in the CA1 region were not due to iron-related oxygen deprivation. 

As a result of iron deficiency, adult (PD70) DNTfR1 mice, with diminished iron uptake, showed impaired spatial learning and memory in the Morris water maze task and structural alterations of apical dendrites in the CA1 region [51]. Further, in earlier development at PD30, iron deficiency due to DNTfR1 expression was associated with a reduced number of cells in CA1 stained for PV and associated with PNNs, compared to same-aged wild-type controls. At PD70, the number of PV-positive cells normalized in CA1 of the DNTfR1 transgene mouse model; however, persistence of iron deficiency was associated with reduced number of PNNs and reduced percentage of PV-positive cells ensheathed with PNNs. Inhibition of DNTfR1 expression in the DNTfR1 transgenic mice starting at PD21, resulting in iron restoration and no behavioral differences observed in the Morris water maze. However, when DNTfR1 inhibition was begun at PD42, deficits in spatial learning and memory persisted into adulthood [51]. This effect of iron depletion due to DNTfR1 on number of PNNs and percentage of PV-positive cells with PNNs was also normalized when iron repletion was begun at PD21 but not PD42 [51]. Finally, DNTfR1 expression and reversible iron deficiency caused significant reduction of expression of the “BDNF-V” splice variant in the CA1 region of the PD70 adult transgene mouse model, which was normalized to control levels when iron repletion was begun at PD21 [51]. In summary, these studies show that the availability of iron stores and local iron uptake is critical for rapid hippocampal development. Iron deficiency during this critical period of fetal and early postnatal development significantly interferes with hippocampal development, which can be largely, if not completely, reversed if iron restoration begins early enough in this critical period and sufficient (local) iron availability is maintained. 

From a clinical perspective, there have been concerns raised in the literature about the prevalence of iron-deficiency anemia and the possible pathogenic role of iron-deficiency in children and adolescents with autistic disorder and ASD [52,53,54,55]. In one study, the iron status of 116 subjects (95 boys, 21 girls; mean age = 8.5 years ±3.6 [SD]) fulfilling DSM-IV criteria for autistic disorder was studied [52]. Subjects with infection or “other inflammatory conditions” and those receiving iron supplementation within the 3-month period prior to assessment and on any dietary restrictions were excluded from the study. Infection and inflammatory conditions were exclusionary criteria because serum ferritin is an inflammatory marker, whose elevation may reflect inflammation and interfere with recognition of iron deficiency [52]. The sample was split between those less than 6 years old and those 6 years old and older. The study results were provocative and worrisome showing that 24.1% (N = 27) of the subjects met criteria for iron-deficiency and 15.5% (N = 18) had actual iron-deficiency anemia. Moreover, a greater percentage of the preschool-age children (32.4%) than school-age children (20.3%) were iron-deficient [52]. The results are, of course, worrisome but if therapeutically effective interventions or prophylactic dietary iron supplementation is to be considered to attenuate autism-related signs and symptoms (including positive effects on cognition and memory), ascertainment of diagnoses and iron-deficiency must occur well before age 3 years. Moreover, with respect to iron status, peripheral measures provide little insight into the quantitative distribution of iron in brain, the efficiency of its uptake into neurons, and local concentrations in the microenvironment of selected neuronal populations, such as those surrounded by PNNs. 

Another study included 100 children and adolescents (N = 84 males), whose ages ranged from 2–18 years (mean age = 8.36 years ± 4.22 [SD]) with an ASD diagnosis according to DSM5 criteria, and a similarly sized control group (N = 100) that was older (mean age = 11.01 years ± 3.73 [SD]) and had fewer males (N = 71) [55]. Infection and inflammatory conditions were exclusionary conditions for both groups. Although prevalence of formally diagnosed iron-deficiency and iron-deficiency anemia did not differ significantly between patient and control groups, peripheral measures of iron status were significantly lower in the patient group. Moreover, when the patient group was stratified according to age (i.e., <6 years and >6 years), peripheral measures of iron status were significantly lower in the preschool-age children with ASD, compared to the subjects with ASD greater than six years of age. Further, among the patients with ASD, patients with intellectual disability had significantly lower values for hemoglobin and hematocrit, and patients formally rated as having “severe” ASD had lower hemoglobin levels. Iron-deficiency anemia was found to be more likely associated with both intellectual disability and severity of ASD. Finally, an inverse relation was found between hematocrit and formal ratings of severity and number of autism-related symptoms [55]. 

Because of concerns about a possible pathogenic relationship between iron-deficiency and ASD, the iron status of a geographically diverse sample of 222 children (87% males; mean age = 5.3 years, ages 2 to 11 years) with ASD, diagnosed according to DSM-IV criteria, was studied in a multi-site cross-sectional study [53]. Only two children met formal criteria for iron-deficiency and only one child met criteria for iron-deficiency anemia. Moreover, serum ferritin levels defined as low (<12 µg/L) were found in only 8% of the children and low transferrin saturation (<10%) was found in only 6% of the sample [53]. The study did not include a marker of inflammation, which could have led to an “under-identification” of low serum ferritin levels. The prevalence of iron-insufficiency in this sample of children and adolescents was similar to that found in the general pediatric population. Clearly, these data suggest that if a pathogenic relationship exists between iron-deficiency and ASD, it is not a robust one [53]. Nonetheless, the brain is at greatest risk from iron-deficiency during a narrow sensitive period in its early development; unfortunately, the iron status of these subjects during the sensitive period of early brain development is not known. Moreover, peripheral measures of iron status may not inform the fast-spiking status of PV expressing neurons nor the maturity of their ensheathed PNNs at the time of the study. 

In summary, during fetal development the creation of optimal functional connectivity may depend on adequate iron stores and “disconnection” can occur due to iron deficiency. Preclinical data support translationally-relevant concerns about iron-deficiency and the dependence of brain development on iron availability during a sensitive period in fetal life and early infancy of rapid hippocampal development. Moreover, based on preclinical data, there is concern that once the sensitive period is closed, iron-replenishment may not fully prevent emergence of long-term adverse effects on recognition memory and cognition in general. Thus, detection and correction of peripheral measures suggestive of reduced iron stores in childhood and adolescence may be too late to prevent long-term functional deficits. As discussed, peripheral measures may give only limited insight into the adequacy of iron distribution and neuronal uptake in brain. Of particular interest is the fact that PV-expression is developmentally delayed in the context of iron deficiency during this critical period and PNN formation is abnormal or does not occur [2]. Synchronous oscillatory output dependent on the input of fast-spiking, PV-expressing GABA inhibitory basket cells onto assemblies of pyramidal output neurons is necessary for functional connectivity between geographically-distant critical nodes comprising critical neural circuits and performance of higher cognitive functions (e.g., working memory) [4,10]. It is hypothesized that as PNNs develop, they play a possible protective role in maintaining a continuous local reservoir of iron (and copper and zinc) in the local microenvironment of selected neurons, which may buffer against at least some fluctuations due to insufficient nutritional iron intake, absorption and distribution to brain [2,56]. The availability of iron supports continued functional connectivity by providing overworked mitochondria in fast-spiking neurons with adequate local stores to support their respiratory activity, while also protecting these neurons against iron-induced oxidative damage [4,10,57,58].

## 4. Copper Homeostasis, Brain Development, and Functional Consequences of Binding to PNNs

Copper ions play familiar biological roles in terms of mitochondrial respiration and ATP production and protection against oxidative stress and damage; moreover, unbound redox reactive copper is itself a mediator of oxidative stress and damage. The transition of unbound copper between its reduced Cu^+^ and oxidized Cu^2+^ states can lead to production of the highly reactive hydroxyl radical (OH)^−^ through the reaction of the superoxide anion (O_2_^−^) and hydrogen peroxide (H_2_O_2_), which can attack proteins, lipids and nucleic acids [1]. Furthermore, unbound copper can play toxic roles by catalyzing interactions of nitric oxide (NO), a gaseous retrograde novel neurotransmitter that regulates receptor-mediated changes in intraneuronal levels of cGMP, with O_2_^−^ or endogenous thiols producing the reactive nitrogen species (RNS) peroxynitrite (CNOO^−^) or S-nitroso-thiols, respectively [1]. Both reactive oxygen species (ROS) and RNS are implicated in the pathogenesis of neurodegenerative diseases, such as Parkinson’s disease (PD), Alzheimer’s disease (AD), and amyotrophic lateral sclerosis (ALS). Thus, the heightened importance of handling the essential copper ion, especially in anatomic areas of brain whose function depends on highly active mitochondrial respiration, such as fast-spiking, PV-expressing GABAergic interneurons in neocortex and hippocampus [4,10,59]. 

Copper levels in brain are estimated to be about 5 µg/g and its estimated concentration in CSF is about 0.3–0.5 µM [1]. Importantly, three copper protein transporters (Ctr1, ATP7A and ATP7B) regulate free copper levels in brain and CSF via transport across specialized polarized endothelial and epithelial cells, respectively, constituting the blood–brain (BBB) and blood/CSF barriers [1,60]. The copper protein transporters are also found more generally in cells within the CNS and periphery, where they work to maintain physiological levels of free copper distributed within distinct cellular and extracellular compartments. The high-affinity, copper transporter, copper transporter 1 (Ctr1), that is not dependent on ATP is located on the apical side of the BBB endothelial cell and works to import free copper from the general circulation [1,60]. Further, Ctr1 may have a regulatory role in maintaining this labile copper signaling pool because neurons isolated from mice with haploinsufficient expression of Ctr1 exhibit higher spontaneous firing. Again, HA and, perhaps, PNNs more specifically, play an important regulatory role in maintaining concentrations of copper and other metal ions in the local microenvironments of neurons [33]. 

The essential nature of copper to neurologic function and the pathological consequences of disturbances of copper levels, distribution and/or binding are highlighted by both Menkes and Wilson’s diseases, which are characterized by mutations in the copper transport genes *ATP7A* and *ATB7B*, respectively [60]. On the apical side of the endothelial cell, ATP7B, a P-type ATPase, transports excess free copper out of the endothelial cell going from the direction of the brain to the general circulation. Wilson’s disease is due to an autosomal homozygous recessive mutation of *ATB7B* located on chromosome 13 [61]. On the basolateral side of the BBB’s endothelial cells, ATP7A, a P-type ATPase that shares about 55% homology with ATP7B, works to transport free copper into the brain parenchyma. ATP7A resides within the membrane of the trans-Golgi network (TGN), where it transports free copper from the cytosol into the lumen of this secretory network for incorporation into cuproenzymes. However, in the event of copper overload, ATP7A is translocated from the TGN in large free copper-containing cytosolic vesicles for incorporation into the plasma membrane and release of excess free copper. In Menkes disease, an X-linked disorder associated with almost 300 loss of function mutations of the gene encoding ATP7A, whose phenotype includes systemic copper deficiency and pleiotropic CNS manifestations, there is cerebrovascular copper accumulation and low brain copper levels [1,60]. Menkes disease highlights the importance of highly regulated handling and transport of copper for CNS function and development; most notably, in Menkes disease, genetic defects in a P-type ATPase causes failures to transport copper across the placenta, GI tract, and BBB. Menkes disease leads to death within the first decade, and demyelination and neurodegeneration are found in cerebral cortex, hippocampus, thalamus and cerebellum [62]. Similarly, acquired copper deficiencies due to diverse etiologies (e.g., excessive zinc consumption, gastrectomy and idiopathic malabsorption) are associated with neurological presentations, primarily affecting the peripheral nervous system. In terms of the blood/CSF barrier, Ctr1 transports free copper from the CSF into the epithelial cell, whereas ATP7A transports free copper into the CSF. ATP7B is located on the other pole of the blood/CSF barrier to transport free copper into blood. 

Because of the potential toxicity of free copper, there are copper chaperone proteins (referred to as metallochaperones) that direct copper to specific targets and pathways through protein-protein interactions (e.g., inserting copper into the active sites of cuproenzymes and transport proteins) [62]. Examples of chaperones include atox1, which transfers copper to P-type ATPases, and cox17, which delivers copper to the mitochondria where it is necessary for assembly of cytochrome C oxidase. P-Type ATPases are not only involved in the directional transport of copper into and out of endothelial and epithelial cells of the BBB and blood/CSF barrier, but are also necessary for compartmentalization of copper within the cell (e.g., transport into the Golgi apparatus for incorporation into secretory proteins and creation of a free pool of releasable copper). 

Once copper has entered the cell, glutathione (GSH) has roles in sequestering copper and, thereby, helping to prevent its toxic accumulation, and its intracellular transport between Ctr1 and copper chaperone proteins [61]. GSH, a soluble tripeptide composed of glutamate, cysteine and glycine, is an antioxidant that acts by itself or as an enzyme cofactor of glutathione peroxidases and glutathione S-transferases to protect against ROS and RNS. In addition to its familiar role to protect against oxidative damage, disturbances of the tightly regulated levels of GSH are associated with pathological disruption of both intracellular copper transport and maintenance of safe nontoxic levels of free copper [61]. For example, an increased ratio of the reduced GSH to glutathione disulfide, the oxidized product resulting from the reaction of GSH with radical species, is observed in differentiated cultured neurons compared to non-differentiated neurons. This equilibrium shift to GSH in differentiated motor neurons is associated with increased ATP7A-dependent copper efflux via the secretory pathway. The bivalent oxidized state of copper interferes with protein degradation by the ubiquitin proteasome system by promoting aggregation and self-oligomerization of ubiquitin, which reduces the pool of free ubiquitin and degradation of misfolded proteins [61]. Moreover, genetic manipulation using RNAi to knockdown GSH biosynthesis in Drosophila caused neuronal defects and embryonic lethality that was attenuated with copper supplementation [61]. Conceivably, pathogenic mechanisms of at least some neurodegenerative diseases could include reductions of cytosolic levels of GSH associated with abnormalities in transport and intraneuronal distribution of copper, and increased intraneuronal levels of Cu^2+^, which, in turn, could result in abnormal protein aggregation, impaired protein degradation, and oxidative damage [61].

Copper also has familiar and more specific CNS roles in catecholamine biosynthesis (dopamine β-hydroxylase) and neuropeptide synthesis (peptidylglycine α-amidating monooxygenase) [1]. Moreover, emerging research shows that copper’s role in CNS extends beyond serving as a cofactor in redox reaction mechanisms. In fact, copper is involved in synaptic transmission, cell-signal transduction cascades, axonal targeting, and formation of neural circuits [1,60,62,63,64]. With respect to synaptic transmission, copper acts both pre- (i.e., vesicular trafficking and interactions between “secretory” proteins) and post-synaptically affecting specific neurotransmitter receptors themselves [1]. Consistent with a proposed role of free copper in synaptic transmission, copper is stored in synaptic vesicles and is released in a depolarization-dependent manner with an estimated minimal concentration in the synaptic cleft as high as 100 µM, which is significantly higher than its concentration in CSF. Physical techniques that use fluorescent small molecule indicators have shown the existence of labile rapidly translocating pools of copper in neurons [64]. Thus, X-ray fluorescence microscopy (XFM) showed a depolarization-dependent, calcium ion-dependent redistribution of copper from the cell body to dendrites. The dependence of this redistribution of copper on calcium signaling is confirmed by its elimination in the presence of BAPTA, a calcium ion chelator. Moreover, chelation and, thereby, depletion of labile pools of endogenous copper are associated with increased neuronal excitability [64]. These data support a depolarization-dependent, calcium ion-dependent mechanism for mobilizing, redistributing and releasing a labile pool of copper that, in turn, modulates excitability in some neurons [64]. In another study, specialized histochemical staining revealed the presence of “labile copper pools” in rat CNS within cell bodies of cortical pyramidal and cerebellar granule neurons, within the neuropil of the cerebral cortex and hippocampus, as well as on the synaptic terminals of locus ceruleus projections [62]. In a voltage-independent manner, exogenous applications of copper inhibited currents generated by stimulation of NMDA, GABA, and glycine receptors [62]. In cultured mouse and rat cortical and hippocampal neurons, copper acted noncompetitively to antagonize NMDA receptor-mediated Ca^2+^ conductance and, thereby, interfere with metabotropic functions of the glutamate/glycine-gated ion channel receptor [62,65]. There are functional electrophysiological consequences of this noncompetitive inhibitory effect of copper on the NMDA receptor shown by the ability of low micromolar copper concentrations to interfere with long-term potentiation in the CA1 region of rat hippocampus [62].

Moreover, an ATP7A-dependent NMDA receptor-mediated release of copper has been reported that links homeostatic regulation of neuronal copper levels with regulation of excitatory neurotransmission [1,62]. Specifically, NMDA receptor-mediated transient increases in intraneuronal Ca^2+^ concentrations are reported to promote rapid and reversible trafficking of ATP7A, the Menkes ATPase, to somatodendritic and axonal compartments of cultured hippocampal neurons. This effect on trafficking of ATP7A was due to NMDA receptor-mediated increases of intraneuronal Ca^2+^ concentrations and shown to be independent of voltage-gated Ca^2+^ entry and generation of action potentials [62]. Moreover, NMDA receptor-mediated trafficking of the Menkes ATPase was associated with the rapid release of copper from hippocampal neurons that was not inhibited by antagonism of voltage-gated Na^+^ channels and, thus, not dependent on propagation of action potentials [62]. The released copper appeared to be free and unbound to cuproproteins as its concentration in the efflux solution following NMDA receptor activation was not reduced by removal and/or precipitation of protein. NMDA receptor-mediated copper efflux involved translocation of a functional Menkes ATPase was shown by the fact it did not occur in mice lacking this specific P-type ATPase. Importantly, NMDA receptor-mediated translocation of the Menkes ATPase creates accumulation of a readily releasable stored pool of copper. This receptor-mediated readily releasable pool of copper may be an endogenous intrasynaptic homeostatic regulatory mechanism of noncompetitively dampening NMDA receptor activation. Although not known with certainty, a proposed mechanism for dampening NMDA receptor activation could be a copper-catalyzed S-nitrosylation of a cysteine residue on the extracellular surface of the NMDA receptor. Further, it is speculated that the increased sensitivity to excitotoxicity, seizures and neurodegeneration observed in Menkes disease may be due, at least in part, to the loss of this noncompetitive intrasynaptic inhibitory effect of copper on excitotoxic NMDA receptor activation as a result of diminished expression of ATP7A [62]. 

Several studies have investigate the potential association of copper levels and a potential biomarker of low zinc/copper ratios in children with ASD [66,67,68]. Plasma levels of copper were reported to be significantly increased in samples of 79 children (68 males; mean age = 11.7 years ± 5.62 [SD]) with autistic disorder and 52 children (47 males; mean age = 9.9 years ± 7.6 [SD]) with pervasive developmental disorder-not otherwise specified (PDD-NOS), diagnosed according to DSM-IV criteria, compared to 18 age and gender similar neurotypical controls [67]. Interestingly, in this study, plasma copper levels did not distinguish the sample of 21 children (19 males; mean age = 14.87 ± 7.87 [SD]) diagnosed with Asperger’s syndrome from the controls. Individual comparisons of plasma levels of zinc within each of the three diagnostic groups with the control sample also did not differ significantly [67]. Preliminary data on plasma levels of copper and zinc were measured but suggest that autism severity and subject age (the mean age of the samples with Asperger’s syndrome was older than the mean ages of the other diagnostic groups) should be considered as possible confounding variables in future studies. As expected, zinc supplementation of the patient samples for a minimum of 8 weeks led to significant increases in plasma zinc levels; unfortunately, supplementation doses and dosing regimen of zinc were not provided nor were the number of subjects treated within each diagnostic group indicated [67]. The authors’ data suggest that zinc supplementation significantly reduced plasma copper levels in the subjects with autistic disorder and PDD-NOS, but did not affect plasma copper levels in the subjects with Asperger’s syndrome. Moreover, symptom severity in the sub-sample of children with autistic disorder receiving zinc supplementation was reported to decrease (however, the autistic children that showed therapeutic benefit also received vitamin B6 supplementation). Again, studies exploring peripheral levels of metals are very hard to interpret because relationships between altered peripheral levels and concentrations within discrete areas of brain are not known. Moreover, the impact of altered metal concentrations in the local microenvironments of specific neurons is very much dependent on the developmental age of the brain; thus, the effect of altered levels may be quite different in preschool-age, school-age and fetal brains. Nonetheless, metals are essential for proper brain development and function [39,55,60,62,64]. Ordinarily, the intact polyanionic character of the HA backbone and CSPGs of PNNs, especially those surrounding the subpopulation of fast-spiking, PV-expressing GABAergic interneurons, may serve as another local mechanism for maintaining divalent metal cations within a narrow range that supports crucial physiological functions, while protecting against a cascade of events with pathogenic consequences [2,4,24,36,69]. 

In summary, the redox reactive nature of the copper ion that cycles between its cuprous (I) and cupric (II) states is the basis of its tightly controlled enzymatic activity; however, when regulatory mechanisms for its transport, distribution and local concentrations fail, elevated concentrations of free redox reactive copper may cause oxidative damage to proteins and lipids. Conceivably, PNNs may participate in regulating and maintaining local concentrations of copper within safe ranges to support metabolic functions, synaptic transmission, neural circuit integrity, as well as providing protection against oxidative damage [4,33,56,62,69]. Importantly, metal cations bind and can displace each other within PNNs that ensheath discrete subpopulations of neurons, suggesting that significant alterations of concentrations within local microenvironments can be expected to have functionally-relevant effects [70]. In addition to its several roles, PNNs may also serve as valuable reservoirs of critical metal ions that buffer against significant fluctuations of concentrations within local microenvironments.

## 5. NMDA Receptor and PNN Function: Effects of Oxidative Stress and Therapeutic Potential

Relative expression of the heterotetrameric GluN2A ionotropic NMDA receptor containing two obligatory GluN1 subunits and two variable GluN2A subunits, compared to the heterotetrameric GluN2B receptor, increases in postnatal life [8,65]. The increased expression of GluN2A receptors on PV-expressing interneurons is linked to functional maturation of these neurons and the PNNs that surround them and cortical network refinement. Mice lacking expression of GluN2A receptors have increased susceptibility to oxidative stress, which is reflected in redox dysregulation and delayed maturation of PNNs [8]. Increased susceptibility to sub-threshold oxidative stress and redox dysregulation, as well as delayed maturation of PV-expressing neurons and their associated PNNs were studied in the anterior cingulate cortex (ACC) of knockout (KO) mice lacking GluN2A expression (GRIN2A KO mice) not exposed and exposed to GBR12909 (GBR; 10 mg/kg, i.p.), a dopamine transporter inhibitor and cause of mild oxidative stress, over postnatal days (PDs) 10 to 20 [8]. At PD20, the intensity of both PV-immunostaining in neuronal processes and histofluorescent-staining of PNNs with *Wisteria floribunda* agglutinin (WFA) in ACC of GRIN2A KO mice was lower, while the number of PV-expressing cells did not differ between GRIN2A KO mice and wild-type mice. At both PD20 and PD60, a significantly lower percentage of PV-expressing cells in the ACC of GRIN2A KO mice were wrapped with PNNs fluorescently-tagged with WFA than PV-expressing cells in the ACC of wild-type mice. 

The GRIN2A KO mice subjected to 10-days of GBR12909-induced mild oxidative stress, which generates ROS, resulted in significantly more intensely-stained 8-oxo-2′-deoxyguanosine (8-oxo-dG) immunoreactivity, a marker of oxidative damage, compared to PBS- and GBR-treated wild-type mice. This mild GBR-induced oxidative stress in the GRIN2A KO mice during PDs 10–20 led to reductions of PV-expressing neurons, PV expression, and less intense WFA-fluorescently-stained PNNs; comparisons were made to PBS- and GBR-treated wild-type mice [8]. The less intense WFA-fluorescently-stained PNNs of the mild GBR-induced oxidatively stressed GRIN2A KO mice may reflect their “chemical” immaturity. Functionally, the chemical maturity of PNNs, which is also reflected in the sulfation patterns of their chondroitin sulfate proteoglycans, is necessary for their sequestration of semaphorin 3A, a chemorepulsive axon guidance protein, and Otx2 (orthodenticle homeobox 2), an important homeobox transcription factor [4,36,41]. The severity of these changes in the GBR-treated GRIN2A KO offspring were attenuated by adding N-acetylcysteine, which possesses antioxidant properties, to the drinking water (2.4 g/L) of the lactating mothers from PDs7 to 20 [8]. 

Effects of GBR-induced mild oxidative insult observed in the ACC persisted in the GRIN2A KO offspring to PD60 [8]. Specifically, in comparisons of the PD60 ACC of GBR-treated GRIN2A KO mice with non-treated KO and wild-type mice, intensity of immunoreactively-stained 8-oxo-dG was significantly greater, and number of PV-expressing cells, PV expression, intensity of WFA fluorescent-labeling, and proportion of PV-expressing cells ensheathed with PNNs were significantly lower [8]. These persisting changes also had persisting functional consequences as reflected in immunochemical markers of microglial activation and reduced “power” of high frequency β-oscillations (12–28 Hz) in ACC slices at PD60 [8].

The heterotetrameric GluN2A receptor is a hypothesized therapeutic target for maintaining synchronous oscillatory output of assemblies of neocortical pyramidal neurons [4,65]. The maturity of PNNs is necessary for differentiation and PV expression of GABAergic interneurons that synchronize this oscillatory output [4,10]. In addition to their roles in refining neural circuitry and closing critical periods, based on their physicochemical properties, PNNs play important roles in protecting against oxidative damage and providing a reservoir of metal and other cations that are critical for NMDA receptor function in the local microenvironment of neocortical neuronal circuits (Figure 3, lower right panel). For example, NMDA receptor function depends on membrane potential-dependent binding of the hydrated magnesium ion to a channel domain of this ionotropic receptor, and binding of zinc contributes to regulation of glutamate-gated channel opening [65,71]. 

The zinc bivalent cation bound to hyaluronic acid can act as an antioxidant and protect hyaluronic acid against oxidative damage by displacing iron ions from their binding sites [33]. Zn^2+^ showed a dose-dependent protective effect against the superoxide radical-induced damage of hyaluronic acid and a “moderate” protective effect against this damage by peroxynitrite. These protective “scavenger” effects of Zn^2+^ were related to its interaction with hyaluronic acid [33]. Importantly, these data show that zinc binds to the hyaluronic acid backbone of the PNN, consistent with the possibility that this binding by the PNN may be relevant to maintenance and fluctuations of local zinc concentrations in the microenvironments of specific neuronal populations and gating of ionotropic NMDA receptors by glutamate and glycine/D-serine. 

Mice with absent or deficient expression of cortactin binding protein 2 (CTTNBP2) (i.e., *Cttnbp2*^-/-^ and *Cttnbp2*^+/-^, respectively), a regulator of dendritic spine formation, show impairments of social preference, social memory and spatial memory, and cognitive inflexibility in standard behavioral paradigms [72]. Interestingly, cells in the CA1 and CA3 regions of the dorsal hippocampus and basolateral amygdala of the *Cttnbp2* knockout mouse showed less-intense C-FOS staining after social stimulation than the wild-type controls. The less-intense C-FOS staining in the *Cttnbp2*^-/-^ mouse was consistent with gene dosage-dependent effects of *Cttnbp2*-deficieny on dendritic spines in the dorsal CA1 hippocampal region and other ultrastructural parameters of the excitatory synapse in dorsal hippocampus, such as the size of the postsynaptic density and number of presynaptic vesicles [72]. *Cttnbp2* deficiency was associated with reduced synaptic distribution of SHANK2 and SHANK3 scaffold proteins, which suggests that CTTNBP2 has an important role in their synaptic targeting; protein levels of SHANK2 and SHANK3 were not reduced in the total homogenates [72]. Moreover, consistent with their roles as scaffold proteins that are involved in anchoring glutamate receptors at the surface of the postsynaptic membrane of excitatory synapses located on dendritic spines, immunoblotting revealed reduced protein levels of the NMDA receptor subunits GRIN1 and GRIN2A in the synaptosomal fractions of *Cttnbp2*^-/-^ brains [72]. Of interest to the current review, zinc binds to and regulates the synaptic distribution of SHANK2 and SHANK3. Further, 7-days of dietary zinc supplementation in drinking water increased the immunoreactive levels of SHANK2, SHANK3, and the GRIN1 and GRIN2B (but not GRIN2A) NMDA receptor subunits in the synaptosomal fraction [72]. Zinc concentrations in *Cttnbp2*^-/-^ brains were lower than those in wild-type littermates, consistent with other data that CTTNBP2 regulates zinc concentration in brain [72]. From a translational therapeutic perspective, deficits of social behavior in *Cttnbp2*^-/-^ mice were ameliorated by administration of either 7-days of dietary zinc supplementation or intraperitoneal D-cycloserine (20 mg/kg) given 30 min prior to testing in a “reciprocal social interaction paradigm” [72]. The data highlight an important role for zinc in the architecture and function of the excitatory synapse located on dendritic spines. Conceivably, PNNs surrounding selective populations of GABAergic and pyramidal neurons participate with CTTNBP2 in this handling and distribution of zinc in the local microenvironment of the excitatory synapse. 

## 6. Discussion

Some neurodegenerative diseases with prominent metal dyshomeostasis include Alzheimer’s disease, Parkinson’s disease, and Friedreich’s ataxia [37]. Rapid scanning X-ray fluorescence spectroscopic imaging (RS-XRF) can simultaneously and non-destructively map and quantify multiple metals in the same section in all chemical forms [37]. This analytic technique is demonstrating pathological changes in metal content and distribution in different neurodegenerative diseases [37]; these studies highlight the presence of iron, copper and zinc dyshomeostasis in these disorders. Algorithms based on the fluorescence escape depth of each element and X-ray density of the brain have been developed that permit expressing concentration in µg/g *w/w*. The resolution allows simultaneous mapping of iron, copper and zinc to discrete regions within the substantia nigra and dentate nucleus of the cerebellum. The technique also permits the quantitative analysis of the effects of specific chelators, some of which are used therapeutically, on the distribution of multiple brain metals in animal models of neurodegenerative diseases. Data showing regionally selective increases in concentration and changes in distribution of metals in neurodegenerative diseases, as well as their binding to disease-related proteins and involvement in pathogenic processes of aggregation and deposition, continue to stimulate therapeutic interest in metal chelators. These therapeutic chelators would not only be non-toxic and able to cross the BBB, but also have selectivity for not only specific ions but also their oxidation states and specific chemical forms. Ideally, chelators will also be found that can selectively target specific brain regions, cells, and subcellular organelles. These imaging strategies explain relationships between iron imbalance and movement disorders because the highest iron concentrations were found in substantia nigra, globus pallidus, putamen, caudate nucleus, red nucleus, dentate nucleus and locus coeruleus. Copper concentrations were also highest in substantia nigra, locus coeruleus, dentate nucleus, and cerebellum; although copper content was also high in basal ganglia, the differences between basal ganglia and other brain regions were not as dramatic as they were for iron. Zinc was more concentrated in the white matter of adult brain than the gray matter, where it has a role in myelin synthesis and stability. Zinc was also concentrated in hippocampus and amygdala [37]. As emphasized throughout this review, metal ions have important regulatory roles and mediate signaling and plasticity in the brain; spatial mapping using these advanced imaging techniques may provide novel insight into mechanism of metal ion localization and the accumulation relevant to disease progression and therapeutic interventions in neurodevelopmental disorders [2,25,63,73]. 

Appreciation of PNNs has evolved from their earlier dismissal as staining artifacts or morphological curiosities without functional relevance to recognition of their critical neurobiological roles, many of which can be explained by their physicochemical properties. Because of their potential toxicities, binding and transport proteins have evolved to maintain free concentrations of iron and copper within narrow physiological ranges. PNNs may serve as both a buffering and “scavenging” mechanism to prevent episodic or sustained toxic concentrations of iron and copper in the microenvironments of the subpopulation of neurons they enwrap, as well as an important local reservoir of these redox reactive metals and other cations. In addition to roles in catalysis at the active sites of metalloenzymes and mitochondrial electron transport, iron is necessary for hippocampal development during a critical period and copper is an important synaptic modulator of NMDA receptor activation. PNNs ensheathing a subpopulation of FSPV+ GABAergic inhibitory interneurons play important roles in maturation and maintenance of their fast-spiking properties, while protecting them against oxidative damage caused by the intense mitochondrial respiratory activity necessary to maintain fast-spiking. Abnormalities of parvalbumin expression and dysregulated GABAergic inhibitory input onto pyramidal output neurons have been described in at least some presentations of autism spectrum disorder. Subpopulations of GABAergic neurons surrounded by PNNs are responsible for the synchronous oscillatory output of assemblies of neocortical pyramidal neurons, the latter output is computed upwards into “rhythms” that functionally connect different areas of the cerebral cortex and underlie higher cognitive functions, such as working memory. Future research will explore therapeutic strategies for remodeling PNNs to increase neural plasticity or improve efficient information transfer by decreasing the “leakiness” of neural circuits; selection of these separate and distinct therapeutic goals will be influenced by developmental age of the patient and pathogenic mechanism(s) of the neuropsychiatric disorder. These therapeutic strategies will almost certainly have to take account of immediate and long-term impact on the “handling” of redox reactive metals and other cations in the local microenvironments of the neurons surrounded by PNNs. To this end, there is emerging interest in identifying and delivering small molecules to cellular elements contributing to PNN architecture and its components that will influence their selective expression of genes coding PNN remodeling enzymes [4]. 

## Abbreviations

8-oxo-dG	8-oxo-2′-deoxyguanosine
ACAN-/-	deletion of aggrecan in homozygous mouse
ACC	anterior cingulate cortex
AD	Alzheimer’s disease
ALS	amyotrophic lateral sclerosis
AP-1	activator protein 1
ASD	autism spectrum disorder
BBB	blood–brain barrier
BCAN-/-	deletion of brevican in homozygous mouse
BDNF	brain-derived neurotrophic factor
Bral2/HAPLN-4	brain link protein
C4S	chondroitin 4 sulfate
C6S	chondroitin 6 sulfate
CNS	central nervous system
Crtl-1/HAPLN-1	cartilage link protein
CSF	cerebrospinal fluid
CSPGs	chondroitin sulfate proteoglycans
CSs	chondroitin sulfates
CTTNBP2	cortactin binding protein 2
DNTfR1	dominant negative nonfunctional transferrin receptor-1
EAE	experimental autoimmune encephalomyelitis
ECM	extracellular matrix
FMRP	fragile X mental retardation protein
FXS	fragile-X-syndrome
GAG	glycosaminoglycan
GSH	glutathione
GSSG	glutathione disulfide
HA	hyaluronic acid
HAPLN1-/-	deletion of cartilage link protein in homozygous mouse
HAS	hyaluronan synthase
KO	knockout
LTP	long-term potentiation
MMP	matrix metalloproteinase
MT	metallothionein
NMDA	N-methyl-D-aspartate
Otx2	orthodenticle homeobox 2
PBS	phosphate buffer solution
PD	Parkinson’s disease
PD	postnatal day
PGs	proteoglycan proteins
PNNs	perineuronal nets
PTR	proteoglycan tandem repeat
PV	parvalbumin
RNS	reactive nitrogen species
ROS	reactive oxygen species
SOD	superoxide dismutase
TfR1	normally-functioning transferrin receptor-1
TGN	trans-Golgi network
TIMP	tissue inhibitor of MMPs
TN-R	tenascin-R
tPA	tissue plasminogen activator
RS-XRF	rapid scanning x-ray fluorescence spectroscopic imaging
WFA	*Wisteria floribunda* agglutinin
XFM	X-ray fluorescence microscopy
